# The effect of parental depression on the problem behaviour and academic performance of children with asthma

**DOI:** 10.1002/nop2.542

**Published:** 2020-06-17

**Authors:** Jeong‐Won Han, Jung Min Kim, Hanna Lee

**Affiliations:** ^1^ College of Nursing Science Kyung Hee University Seoul Korea; ^2^ Department of Nursing Science Jinju Health College Jinju Korea; ^3^ Department of Nursing Gangneung‐wonju National University Gangneung Korea

**Keywords:** asthma, depression, nurses, nursing, problem

## Abstract

**Aim:**

This study attempts to determine the factors affecting problem behaviour and academic performance of children with asthma.

**Design:**

This is a cross‐sectional study used actor and partner interdependence model.

**Methods:**

Data of 236 children and their parents from the 10th Panel Study on Korean Children were used, and analysis was performed using SPSS 20.0 and AMOS 20.0. We have received panel data from the Korea Institute of Child Care and Education on April 10th, 2019.

**Results:**

Parental depression had actor and partner effects on marital conflict, and marital conflict perceived by the father had actor and partner effects on parent–child interactions, while marital conflict perceived by the mother had only actor effect on parent–child interactions. Parent–child interaction perceived by both fathers and mothers was found to affect the problem behaviour of children. The problem behaviour of children affects academic performance.

## 1 INTRODUCTION

Recently, the number of patients complaining of respiratory diseases due to particulate matter has been increasing; more specifically, dust has been reported to seriously affect the health of children with asthma (Orellano, Quaranta, Reynoso, Balbi, & Vasquez, 2018). In many cases, asthma begins in early childhood (43.2% of patients with asthma are below the age of 12) and continues to adulthood (National Health Insurance Corporation, 2016). The health issues of children with asthma are emerging as a social problem as asthma often requires repetitive and long‐term treatment that places a significantly higher financial burden on parents raising children with asthma (Everhart, Greenlee, Winter, & Fiese, 2018). Furthermore, parents of children with asthma have the psychological burden of performing multiple roles since they simultaneously experience the disease process and growth of their children (Taminskiene et al., 2019).

Experiencing financial and psychological burdens at the same time increases the probability of experiencing depression for parents of children with asthma. Their depression is an important factor connected to the recovery and school life of their children (Endrighi, McQuaid, Bartlett, Clawson, & Borrelli, 2018). Depression of parents who raise children with asthma is a mental health issue that affects both parents; if one of the parents experiences depression, the other will be affected too, resulting in issues such as marital conflict, which will negatively affect the relationship between parents and children, as well as children's social lives (Endrighi et al., 2018). Accordingly, research on children with asthma and their parents needs to collect data on parents and children as a unit and make efforts to understand the phenomena by analysing the data of parents using actor and partner interdependence model. Since married couples are in an interdependent relationship, the Actor‐Partner Interdependent Model (APIM) proposed by Kenny (1996) is used to analyse the interrelationships between variables related to parental depression. In APIM, the effect of an independent variable on its dependent variable is called an actor effect and the effect of an opponent's independent variable on its dependent variable is called a partner effect. If couples' data are separately analysed, interpersonal dynamics between the couple cannot be examined. Even if data were collected from all couples and interdependent data were analysed by treating them as independent data, it would violate one of the main assumptions of inferential statistics, that is data independence, resulting in smaller estimated standard errors and possibly Type I errors. Accordingly, this study attempted to compile basic data to develop a programme for children with asthma and their parents. Using children with asthma and their parents as subjects, the study attempted to determine the relationship between children's problem behaviour and performance in school based on actor and partner effects that are outcomes of parental depression, marital conflict and interactions between children and parents.

## 2 BACKGROUND

Recent research has reported that the relationship between parents and children is closely related to children's growth and development (Feinberg, 2003). From an ecological perspective, parents and children are members of the family system (Crnic & Low, 2002); therefore, they are connected as a unit. Consequently, it is important to approach children's health problems by understanding parents' concerns and the familial status. In particular, parents' emotions are affected by spouses' emotion status (Barnett, Deng, Mills‐Koonce, Willoughby, & Cox, 2008). This crossover effect in the family system indicates how interactions among family members affect the emotions of other members (Han & Lee, 2020). Moreover, parents' emotions can affect children's relationships, growth and development (Barry & Kochanska, 2010).

A previous study of children with asthma (Brown et al., 2006) reported that parental depression is related to their medical institution visits, emergency room visits, hospitalizations and asthmatic complications. Some studies (Kim‐Cohen, Moffitt, Taylor, Pawlby, & Caspi, 2005; Turney, 2012) have reported that parental depression increases incidence of parental neglect, which negatively affects children's recovery. Children with asthma are highly dependent on parents due to the disease, and since school‐going children grow through close interaction with their parents, their experience of an unstable parent–child relationship may cause them to display problem behaviour such as depression, anxiety, withdrawal behaviour and sleep disorders (Oh et al., 2018).

The repeated display of problem behaviour of school‐going children tends to be sustained and reinforced as children grow and such children will eventually experience maladjustment to school life and decreased academic competence. A previous study (Kaugars, Klinnert, & Bender, 2004) on children with asthma confirmed that family factors are among those that have the greatest influence on children's adjustment to daily life. Negative emotions of parents negatively influence interactions between parents and children, ultimately causing problem behaviour in children (Wolf, Miller, & Chen, 2008). These factors have been found to reduce learning flow and performance of children at school. Eventually, parents' mental health and the relationship between parents and children become crucial factors that affect not only children's health but also their adjustment and performance at school (Lim, Wood, Miller, & Simmens, 2011; Wolf et al., 2008). Accordingly, in addition to managing the health of children with asthma, it is necessary for nurses to address the mental health of parents to improve their children's recovery and comfortable adjustment to school life, thereby also enhancing children's performance at school.

## 3 METHOD

### 3.1 Design

The purpose of this study was to investigate the effects of parental depression, marital conflict and parent–child interaction on the behavioural behaviour (internalization and externalization) and academic performance of parents raising 9‐year‐old children with asthma symptoms. A cross‐sectional research study used children's panel data. This study used a actor and partner interdependence model of parental variables.

### 3.2 Participants and procedures

The subjects of the present study were children and parents who participated in the 10th annual (2017) Panel Study on Korean Children (Korea Institute of Child Care and Education, KICCE). The Panel Study on Korean Children that is used in the present study is a longitudinal survey of children born in 2008, their parents and the community environment. Currently, data up to the 10th year (2017) of the Panel Study on Korean Children are released to the general public. The present study used the data of parents who were 19 years or older and their children.

The Panel Study on Korean Children targeted households that had given birth to a newborn baby from April to July 2008 at the sample medical institutions that had 500 or more annual deliveries in 2006. It excluded households not included in the sample survey and the ones who declined to participate in the survey. Other subjects excluded from the survey were cases where the mother of a newborn baby was not able to communicate in Korean, the mother's postnatal health was very poor, the newborn or mother had a serious illness, the newborn was expected to be adopted, the mother had given birth to more than one child, and the mother was 18 years or younger. The Panel Study on Korean Children recruited a preliminary sample of 2,562 households of which 2,150 households with a newborn were selected as the final sample. Sampling of the Panel Study on Korean Children employed a stratified multi‐stage sampling technique where birthing medical institutions were selected in the 1st stage, households who gave birth at the selected medical institutions as a pilot sample in the 2nd stage, and the final sample was drawn from the pilot sample households that were willing to participate in the panel survey in the 3rd stage. The sample maintenance rate presented by the research team of the Panel Study on Korean Children (69% for the 10th panel survey) was confirmed for the sample validity of the present study. Among those who participated in both panel survey and health survey, the final subjects selected for the present study were 236 children who experienced asthma symptoms and their parents, that is 236 fathers and 236 mothers.

### 3.3 Data collection and analysis

The data of this study were provided by the Panel Study on Korean Children through their homepage (http://panel.kicce.re.kr). In addition, the construct and correlations and multicollinearity of measurement variables were determined by Pearson correlation coefficients and the reliability of instruments was confirmed using Cronbach's *α*. The measurement invariance test was conducted to determine whether the data of mothers and fathers had homogeneity in the measurement instrument. The maximum likelihood method was used to test the goodness of fit of the model, and confirmatory factor analysis was performed to confirm the validity of latent variables. The evaluation of the goodness of fit of the model was confirmed using *χ* ^2^, *χ* ^2^/*df*, RMSEA, SRMR, GFI, AGFI, CFI, NFI or Tucker–Lewis Index (TLI), which are absolute goodness‐of‐fit indices. The statistical significance of direct effect, indirect effect and total effect was determined using bootstrapping. Test of structural model invariance across the groups is an analysis technique that studies path coefficients between measurement models and analysis for the study was conducted through the process of metric invariance constraints and cross‐group equality constraints.

### 3.4 Ethical consideration

This study uses secondary data. The 10th Korean Children Panel Survey was conducted after a review by the Institutional Review Board of KICCE(IRB No. KICCEIRB‐2017‐05); the parents provided written consent to their children's participation in the panel survey. This study was also reviewed by the Ministry of Health and Welfare's Public Institutional Review Board (IRB No. P01‐201904‐23‐002).

### 3.5 Measurement

#### 3.5.1 Depression

Depression was measured with an abbreviated depression scale (K6) consisting of six items developed by Kessler et al. (2002) for the U.S. National Health Interview Survey (NHIS) and to measure the mental health of the general public. Each item was measured on a 5‐point Likert scale: no feeling at all (1 point), not feeling very much (2 points), often feeling (3 points), almost feeling (4 points) and always feeling (5 points), with a higher score indicating a higher level of depression. Depression is classified as normal for the range 6–13 points, mild/moderate for 14–18 points and severe for 19–30 points. The reliability of the instrument measured by Cronbach's *α* in the study of Kessler et al. (2002) was 0.89. In the current study, Cronbach's *α* for the fathers' and mothers' instruments was 0.93 and 0.92, respectively.

#### 3.5.2 Marital conflict

The marital conflict scale was developed by Markman, Stanley, and Blumberg (2001) and translated and modified by the research team of the Panel Study on Korean Children. It consists of a total of eight items measured on a 5‐point scale: strongly disagree (1 point), disagree (2 points), neutral (3 points), agree (4 points) and strongly agree (5 points). The reliability of the instrument measured by Cronbach's *α* was 0.96 in the study of Markman et al. (2001), and in the current study, it was 0.91 (father) and 0.92 (mother).

#### 3.5.3 Parent–child interaction

For parent–child interaction, the research team of the Panel Study on Korean Children extracted a few items asking about parent–child interactions based on the home environment, activities and cognitive stimulation from the Early Childhood Longitudinal Study‐Kindergarten cohort (ECLS‐K). The research team of the Panel Study on Korean Children obtained permission to use the instrument from the ECLS research institute and used it as the final measuring instrument after the confirmation of reverse translated content by a third party from the ECLS. The instrument consists of a total of nine items on a 4‐point scale: never (1 point), once or twice a week (2 points), 3–6 times a week (3 points) and every day (4 points); items were measured by parents. The higher the sum of the scores, the higher the interaction between parent and child. Cronbach's *α* for father's and mother's instruments in the study was 0.89 and 0.82, respectively.

#### 3.5.4 Problem behaviour of children

To measure problem behaviour in the survey of the Panel Study on Korean Children, the behaviour evaluation scale of the Child behaviour Checklist (CBCL) developed by Kim, Lee, Moon, Kim, and Oh (2009) was used. For the current study, questions related to internalizing and externalizing problems were extracted and used. Internalizing problems refer to internalized and overly controlled behaviour, such as passive and withdrawn behaviour, emotional insecurity and physical symptoms, and externalizing problems refer to attention problems and less controlled behaviour such as aggressive behaviour. The instrument consists of a total of 100 items measured on a 3‐point scale: never (0 point), sometimes (1 point) and often (2 points); these items are measured by the child. The higher the score, the higher the level of problem behaviour. The reliability of the instrument measured by Cronbach's *α* was 0.77–0.86 in the study of Kim et al. (2009), and the reliability of the instrument in the study was 0.80–0.82.

#### 3.5.5 Academic performance of children

The academic performance of children refers to the results of a longitudinal effect study of comprehensive childcare services of Samsung Childcare Center by Rhee, Lee, Kim, and Jun (2010), which was presented by the research team of the Panel Study on Korean Children. Academic performance scale is composed of a total of 10 items [5‐point scale: not yet (1 point), beginning (2 points), in progress (3 points), intermediate (4 points), proficient (5 points), not applicable (0 point)] in three areas: four items of Korean language; five items of mathematics; and one item of overall competence on school performance. This scale was measured by a web‐based questionnaire and the teacher in charge of the child at school. Cronbach's *α* reliability coefficient in the current study was 0.98.

## 4 RESULTS

### 4.1 General characteristics of the subjects

The average ages of fathers and mothers were 40.1(*SD* 3.5) years and 37.6(*SD* 3.6) years, respectively. In total, 89 (37.7%) fathers had only a high school diploma, 51 (21.6%) were college graduates, and 96 (40.7%) had a bachelors or higher degrees. Regarding mothers' education, 59 (25.0%) had only a high school diploma, 89 (37.7%) were college graduates, and 88 (37.3%) had bachelors or higher degrees. In terms of occupation, 107 (45.3%) of the fathers were managers or held white‐collar jobs, while 115 (48.7%) mothers were full‐time housewives and 81 (34.3%) were managers or held white‐collar jobs. The average household income was KRW 464 million. Totally, 140 (59.3%) children were male and 96 (40.7%) female.

### 4.2 Correlations between measurement variables

Each measured variable was found to be normally distributed with the absolute values of skewness (0.59–0.79) and kurtosis (−0.83–1.91) of less than two and four. All variables had statistically significant correlations at the significance level of 0.05, and no multicollinearity was found among the variables since the absolute value of correlation coefficients among the variables was no greater than 0.8 (Table 1).

**TABLE 1:** Correlation of variables

Variables	X1	X2	X3	X4	X5	X6	X7	X8	X9
X1: Depression (Father)	1								
X2: Depression (Mother)	35*	1							
X3: Marital conflict (Father)	53*	0.36*	1						
X4: Marital conflict (Mother)	30*	0.54*	0.63*	1					
X5: Parent–child interaction (Father)	−0.13*	−0.10*	−0.29*	−0.20*	—				
X6: Parent–child interaction (Mother)	−0.16*	−0.16*	−0.19*	−0.13*	0.24*	1			
X7: Internalizing problem behaviour (Child)	0.11*	0.17*	0.18*	0.18*	−0.16*	−0.13*	1		
X8: Externalizing problem behaviour (Child)	0.14*	0.28*	0.12*	0.19*	−0.15*	−0.12*	0.22*	1	
X9: Academic performance (Child)	−0.18*	−0.31*	−0.13*	−0.27*	0.13*	0.19*	−0.28*	−0.69*	1

Note

* p < .05.

### 4.3 Measurement invariance test

Measurement invariance test was conducted to determine whether the data of paternal and maternal depression, marital conflict and parent–child interaction had homogeneity in the measurement instrument, and four competing models were compared. The first model was a default model. For the second model, factor coefficients were restricted; for the third, error covariances were restricted; and for the fourth, factor coefficients and error covariances were restricted. Metric invariance tests, which were conducted to compare the goodness of fit using *χ* ^2^, TLI, CFI and RMSEA (indices that are not sensitive to the number of cases), determined that metric invariance was secured (Table 2).

**TABLE 2:** The test of measurement equivalence

	Model	χ^2^	*df*	TLI	CFI	RMSEA
Depression						
Model 1	Unconstrained model	119.00	53	0.92	0.93	0.06
Model 2	Measurement weights constrain	129.73	61	0.92	0.93	0.05
Model 3	Measurement residual constrain	91.89	43	0.91	0.93	0.05
Model 4	Measurement weights and residual constrain	109.89	63	0.91	0.93	0.05
Marital conflict						
Model 1	Unconstrained model	269.36	103	0.93	0.94	0.04
Model 2	Measurement weights constrain	335.45	117	0.89	0.90	0.05
Model 3	Measurement residual constrain	213.50	95	0.94	0.95	0.03
Model 4	Measurement weights and residual constrain	273.59	109	0.90	0.91	0.05
Parent–child interaction						
Model 1	Unconstrained model	352.30	169	0.92	0.92	0.03
Model 2	Measurement weights constrain	378.24	178	0.89	0.90	0.03
Model 3	Measurement residual constrain	303.72	159	0.90	0.92	0.01
Model 4	Measurement weights and residual constrain	340.45	179	0.90	0.91	0.04

### 4.4 The effect of parental variables on children's problem behaviour and academic performance

To test the effect of parental depression, marital conflict and parent–child interaction on children's problem behaviour and academic performance, the normality of measurement variables was tested. The univariate normality of each measurement variable satisfied normality assumptions with the absolute values of skewness and kurtosis being ≥2. However, the assumptions of multivariate normality were not satisfied, since the multivariate kurtosis index was at 16.26 and critical ratio (C.R) at 9.26 at significance level of 0.05. If multivariate normality is not satisfied, a problem of upward skewing of the critical value can occur when parameters are estimated. However, studies have shown that even if multivariate normality is not achieved, if the sample size is 120 or higher, maximum likelihood method can be used to estimate parameters. Based on these reports, models were estimated without transforming the data. The goodness of fit of hypothetical models was evaluated using GFI, AGF, CFI, NFI, TLI, RMSEA and SRMR. If GFI, AGFI, CFI, NFI and TLI are 0.90 or higher, the goodness of fit of the model is considered to be favourable. For RMSEA and SRMR, if the value is smaller than 0.05, it is considered an indicator of good fit, while values between 0.05–0.10 indicate an average fit and values of 0.10 or higher indicate low goodness of fit. Hypothetical models of this study were tested using the maximum likelihood method, and the results were *χ* ^2^ = 76.20, *df* = 30, RMSEA = 0.04, SRMR = 0.05, GFI = 0.94, AGFI = 0.92, CFI = 0.93, NFI = 0.92 and TLI = 0.92, which indicated that the hypothesized data fit the model well, confirming the model. Fourteen out of a total of 18 hypotheses were retained in this study (Table 3).

**TABLE 3:** The effect of parental variables on children's problem behaviour and academic performance

Independent variables	Dependent variables	*β*	*B*	*SE*	C.R	*p*	*Direct effect*		*Indirect effect*		*Total effect*	
							*β*	*p*	*β*	*p*	*β*	*p*
FD	→FMC	0.46	0.46	0.05	8.04	<.001	0.46	<.001	—	—	0.46	<.001
MD	→FMC	0.21	0.20	0.06	8.42	<.001	0.21	<.001	—	—	0.21	<.001
FD	→MMC	0.14	0.15	0.06	2,37	.018	0.14	.018	—	—	0.14	.018
MD	→MMC	0.49	0.52	0.06	8.42	<.001	0.49	<.001	—	—	0.49	<.001
FMC	→FPCI	−0.27	−0.19	0.05	−3.39	<.001	−0.27	<.001	—	—	−0.27	<.001
MMC	→FPCI	−0.13	−0.12	0.05	−1.41	.068	−0.13	.068	—	—	−0.13	.068
FD	→FPCI	—	—	—	—	—	—	—	−0.13	.012	−0.13	.012
MD	→FPCI	—	—	—	—	—	—	—	−0.17	.035	−0.17	.035
FMC	→MPCI	−0.12	−0.19	0.05	−3.39	<.001	−0.12	<.001	—	—	−0.12	<.001
MMC	→MPCI	−0.15	−0.13	0.05	−2.60	.047	−0.15	.047	—	—	−0.15	.047
FD	→MPCI	—	—	—	—	—	—	—	−0.15	.027	−0.15	.027
MD	→MPCI	—	—	—	—	—	—	—	−0.10	.049	−0.10	.049
FPCI	→IPB	−0.16	0.15	0.06	−2.07	<.001	−0.16	<.001	—	—	−0.16	<.001
MPCI	→IPB	−0.18	0.37	0.06	−3.21	<.001	−0.18	<.001	—	—	−0.18	<.001
FMC	→IPB	—	—	—	—	—	—	—	0.10	.042	0.10	.042
MMC	→IPB	—	—	—	—	—	—	—	0.10	.007	0.10	.007
FD	→IPB	0.11	0.07	0.04	1.17	.064	0.11	.064	0.01	.002	0.12	.028
MD	→IPB	0.26	0.61	0.04	3.87	<.001	0.26	<.001	0.04	.008	0.31	.006
FPCI	→EIP	−0.13	0.36	0.06	−0.52	.601	−0.13	.601	—	—	−0.13	.601
MPCI	→EIP	−0.16	0.17	0.06	−2.49	.013	−0.16	.013	—	—	−0.16	.013
FMC	→EIP	—	—	—	—	—	—	—	0.12	.007	0.12	.007
MMC	→EIP	—	—	—	—	—	—	—	0.19	.025	0.19	.025
FD	→EIP	0.14	0.20	0.04	1.52	.102	0.14	.102	0.12	.030	0.26	.011
MD	→EIP	0.30	0.18	0.04	4.62	<.001	0.30	<.001	0.13	.040	0.43	.011
FD	→APA	—	—	—	—	—	—	—	−0.16	.042	−0.16	.042
MD	→APA	—	—	—	—	—	—	—	−0.18	.007	−0.18	.007
FMC	→APA	—	—	—	—	—	—	—	−0.13	.031	−0.13	.031
MMC	→APA	—	—	—	—	—	—	—	−0.12	.038	−0.12	.038
FPCI	→APA	—	—	—	—	—	—	—	0.18	.034	0.18	.034
MPCI	→ APA	—	—	—	—	—	—	—	0.24	.009	0.24	.009
IPB	→APA	−0.16	0.11	0.01	2.01	.033	−0.16	.033	—	—	−0.16	.033
EIP	→APA	−0.23	0.35	0.01	3.68	<.001	−0.23	<.001	—	—	−0.23	<.001

Note

APA, Academic performance; C.R, Critical ratio; EIP, Externalizing problem behaviour; FD, Father's depression; FMC, Father's marital conflict; FPCI, Fathers' parent–child interaction; IPB, Internalizing problem behaviour; MD, Mother's depression; MMC, Mother's marital conflict; MPCI, Mothers’ parent–child interaction; *SE*, Standard error.

Fathers' and mothers' depression had an actor effect of *β* = 0.46 (*p* < .001) and *β* = 0.49 (*p* < .001), respectively, and a partner effect of *β* = 0.14 (*p* = .018) and *β* = 0.21 (*p* < .001), respectively, in marital conflict. Marital conflict of fathers had an actor effect (*β* = −0.27, *p* < .001) and a partner effect (*β* = −0.12, *p* < .001) in parent–child interaction, while marital conflict of mothers had an actor effect (*β* = −0.15, *p* = .047) in parent–child interaction. The parent–child interaction of fathers and mothers had a direct effect of *β* = −0.16 (*p* < .001) and *β* = −0.18 (*p* < .001), respectively, on the internalizing problem behaviour of children. Mothers' parent–child interaction was found to have a direct effect (*β* = −0.16, *p* = .013) on the externalizing behaviour problem of children. In addition, mothers' depression was found to have a direct effect on internalizing (*β* = 0.26, *p* < .001) and externalizing behaviour problems (*β* = 0.30, *p* <. 001). Furthermore, parental depression and marital conflict were found to have an indirect effect on internalizing and externalizing behaviour problems. The internalizing problem behaviour (*β* = −0.16, *p* = .033) and externalizing problem behaviour (*β* = −0.23, *p* < .001) were found to have a direct effect on children's academic performance and parental depression, and marital conflict and parent–child interaction were found to have an indirect effect on children's academic performance.

### 4.5 Test of group differences according to household income

To identify significant differences of path coefficients between groups of more and less than average household incomes of subjects, the critical ratios for the difference between free and constrained models for the 18 paths existing in the research models were determined. The results showed a statistically significant difference in the path where mothers' depression influences fathers' marital conflict (critical ratio for difference = 2.27, *p* < .05) (Table 4). The standardized path coefficient from mothers' depression to fathers' marital conflict in the group that earned less than the average income was *β* = 0.43 (*p* < .001), while that of the group that earned more than the average income was *β* = 0.07 (*p* = .059).

**TABLE 4:** The homogeneity test of coefficients applying an invariance constraint for each path

Model		Critical ratio for difference
Model 1	Depression (Father) ‐> Marital conflict (Father)	0.36
Model 2	Depression (Father) ‐> Marital conflict (Mother)	−0.63
Model 3	Depression (Mother)‐> Marital conflict (Father)	2.27*
Model 4	Depression (Mother)‐> Marital conflict (Mother)	0.34
Model 5	Marital conflict (Father) ‐> Parent–child interaction (Father)	−0.15
Model 6	Marital conflict (Father) ‐> Parent–child interaction (Mother)	1.03
Model 7	Marital conflict (Mother) ‐> Parent–child interaction (Father)	−0.54
Model 8	Marital conflict (Mother) ‐> Parent–child interaction (Mother)	1.47
Model 9	Parent–child interaction (Father) ‐> Internalizing problem behaviours	−0.19
Model 10	Parent–child interaction (Father) ‐> Externalizing problem behaviours	−1.11
Model 11	Parent–child interaction (Mother) ‐> Internalizing problem behaviours	−1.41
Model 12	Parent–child interaction (Mother) ‐> Externalizing problem behaviours	−1.93
Model 13	Depression (Father) ‐> Internalizing problem behaviours	−0.84
Model 14	Depression (Father) ‐> Externalizing problem behaviours	−0.30
Model 15	Depression (Mother)‐> Internalizing problem behaviours	−0.40
Model 16	Depression (Mother)‐> Externalizing problem behaviours	−0.51
Model 17	Internalizing problem behaviours ‐> Academic performance ability	−1.76
Model 18	Externalizing problem behaviours ‐> Academic performance ability	0.84

* *p*<.05.

## 5 DISCUSSION

This study was conducted using children with asthma and their parents as subjects to determine the actor and partner effects of parental depression, marital conflict and interaction between children and parents on children's problem behaviour and academic performance.

First, the results showed that parental depression has actor and partner effects on marital conflict. This finding is consistent with the results of an earlier study that reported that parental depression affects marital conflict and negatively affects children's physical and emotional development (Hanington, Heron, Stein, & Ramchandani, 2012). Depression is a negative emotion with mutual transfer phenomena, and the transfer phenomenon of depression can be strong and rapid in relationships where daily stress is experienced in the same space where parents raise children (Thompson & Bolger, 1999). In particular, because parents of children with asthma have a greater psychological burden of performing many roles as parents since they continuously experience the disease process and growth of children at the same time (Taminskiene et al., 2019), the transfer phenomenon of parental depression is strong and fast and ultimately becomes the cause of marital conflict, which in turn negatively influences the growth and development process, resulting in problem behaviour and affecting the academic performance of children. Since this study found that maternal depression has a direct influence on children's internalizing and externalizing problem behaviour, the effect of maternal depression on children appears to be greater than that of paternal depression. Therefore, it is important for nurses who care for children with asthma to periodically access the parents' depression as well. Nurses must make efforts to provide positive impetus for children's growth and development by helping parents maintain a positive relationship with each other. This may be done by including programmes that reduce parental depression in the process of managing children's asthma. In addition, the results of the hypothesis test according to household incomes showed that the influence of maternal depression on fathers' marital conflict was found to be high in the group that earns less than the average income. The reason for this is that the level of depression of mothers in families of lower income may be relatively higher, as asthma requires repetitive and long‐term treatment, placing a financial burden on parents raising children with asthma, which may be particularly felt by families of lower income. It also influences fathers who are responsible for the financial activities of the family. Accordingly, it is necessary for the government to prepare a financial support system for families raising children with asthma. Nurses must also develop nursing plans and interventions for parents and children with asthma in low‐income families and find ways to help them regarding the community.

Second, marital conflict perceived by fathers had actor and partner effects on parent–child interactions, while marital conflict perceived by mothers had an actor effect on parent–child interactions. These results are similar to those of a study on Italian parents and children (Camisasca, Miragoli, & Di Blasio, 2016), which found that marital conflict is a factor that influences parent–child interaction. In light of the emotional security theory, the finding indicates that if the relationship between parents is negative, especially if marital conflict is high, children will perceive their home as an environment that threatens their safety, which has an impact on parent–child interaction negatively (Davies & Cummings, 1994). In particular, since the importance of the father in the family is greatly emphasized in South Korea, marital conflict perceived by fathers appears to negatively influence parent–child interaction perceived by mothers. Accordingly, nurses must develop a mother–father interaction programme that can increase interactions between parents and children by reducing marital conflict. Such programmes will assure children with asthma that they are receiving appropriate treatments and their environment as safe.

Third, parent–child interaction perceived by fathers was found to affect the internalizing problem behaviour of children and parent–child interaction perceived by mothers was seen to affect the internalizing and externalizing problem behaviour of children. These findings are similar to that of a study on US mother–child, which reported that interaction between the mother and child affects the problem behaviour of children (Combs‐Ronto, Olson, Lunkenheimer, & Sameroff, 2009). Since the interaction between fathers and children has a significant role to play in the socialization of children, the parent–child interaction perceived by fathers appears to influence the internalizing problem behaviour in children, such as passive and withdrawn behaviour, emotional insecurity and physical symptoms. Mothers, however, appear to affect the internalizing and externalizing problem behaviour of children due to their strong emotional interaction with children. Therefore, it is necessary for nurses to create nursing programmes and management systems that can maintain positive interaction between parents and children to prevent the problem behaviours in children with asthma.

Fourth, the findings of this study indicate that children's problem behaviour influences their academic performance, similar to the findings of a longitudinal study conducted on children in the United States (Kremer, Flower, Huang, & Vaughn, 2016). It appears that children who display problem behaviour repeatedly have low academic performance because children's problem behaviour negatively influences concentration and memory abilities that are related to learning. Because children with asthma have limited physical activities and peer relations at school due to their illness and possibly have a low attendance rate due to repeated treatments, it is important for them to reduce problem behaviour so that they can adjust to school life well. Therefore, it is necessary for nurses working in schools and medical institutions to observe problem behaviour in children with asthma and improve their adjustment to school by preventing the decline of their academic performance through early identification and management of problem behaviour. This study is significant because it offers basic data for the improvement of academic performance of children with asthma by studying parents' psychological variables, children's problem behaviour and their academic performance at the same time.

## 6 CONCLUSION

This study consisted of a cross‐sectional descriptive survey using the data of the Panel Study on Korean Children, conducted to determine the influence of the depression, marital conflict and parent–child interaction of parents on problem behaviour and academic performance of asthmatic children. The study is significant because it prepares basic data for the improvement of academic performance of children with asthma by studying parents' psychological variables, children's problem behaviour and their academic performance at the same time. Since psychological variables are not formed in a short time, future research that longitudinally confirms the influence using variables presented in the present study is necessary. Furthermore, future studies will require analysis, including other factors considered to affect problem behaviour and academic performance.

## CONFLICT OF INTEREST

The authors declare that they have no conflict of interest.

## AUTHORS' CONTRIBUTIONS

HJW developed a hypothesis, searched the literature, reviewed the relevant articles, analysed the data, interpreted the findings, and wrote a manuscript. KJM and LH developed the hypothesis, reviewed the relevant article, and wrote the manuscript. All authors have read and approved the manuscript.

## CONSENT TO PARTICIPATE

The survey received written informed consent from study participants.
